# Neuropsychiatric lupus erythematosus with neurogenic pulmonary edema and anti-α-amino-3-hydroxy-5-methyl-4-isoxazolepropionic acid receptor limbic encephalitis: a case report

**DOI:** 10.1186/s12883-022-02747-6

**Published:** 2022-06-17

**Authors:** Rongqi Li, Yingai Wang, Xiuhua Wu, Junping Wang, Wei Wei, Xin Li

**Affiliations:** 1 Department of Rheumatology and Immunology, Tianjin Medical University General Hospital, No.154 Anshan Road, Tianjin, 300052 China; 2 Department of Radiology, Tianjin Medical University General Hospital, No.154 Anshan Road, Tianjin, 300052 China

**Keywords:** Systemic lupus erythematosus, Neuropsychiatric lupus erythematosus, Neurogenic pulmonary edema, Anti-α-amino-3-hydroxy-5-methyl-4-isoxazolepropionic acid receptor

## Abstract

**Background:**

Systemic lupus erythematosus (SLE) is a chronic multisystem autoimmune inflammatory disease predominantly found in women of child-bearing age. Neurogenic pulmonary edema (NPE) is a recalcitrant complication that occurs after injury to the central nervous system and has an acute onset and rapid progression. Limbic encephalitis is an inflammatory encephalopathy caused by viruses, immune responses, or other factors involving the limbic system. NPE caused by SLE is rare.

**Case presentation:**

Here, we report a case of a 21-year-old woman with SLE who experienced five episodes of generalized tonic–clonic seizure after headache and dyspnea. Anti-α-amino-3-hydroxy-5-methyl-4-isoxazolepropionic acid receptor (AMPAR) 2 antibody was tested positive in the serum and cerebrospinal fluid. Electrocardiography (EEG) indicated paroxysmal or sporadic medium amplitude theta activity. In addition, chest computed tomography (CT) showed multiple diffuse consolidations and ground-glass opacities. We finally considered a diagnosis of NPE and AMPAR limbic encephalitis. The patient's symptoms improved obviously after methylprednisolone pulse therapy and antiepileptic treatment.

**Conclusions:**

NPE can be a complication of neuropsychiatric lupus erythematosus (NPSLE). AMPAR2 antibodies may be produced in NPSLE patients, especially in those with high polyclonal IgG antibody titers. More basic and clinical studies are required to confirm these observations and elucidate the pathogenicity of encephalitis-related autoantibodies in SLE patients.

## Background

Systemic lupus erythematosus (SLE) is an autoimmune disease characterized by multiple organ damage. Neuropsychiatric lupus erythematosus (NPSLE) is diagnosed when the nervous system is involved in patients with SLE. The incidence of neurological complications in SLE is 21%–95% [1]; this condition has a poor prognosis. Anti-α-amino-3-hydroxy-5-methyl-4-isoxazolepropionic acid receptor (AMPAR) limbic encephalitis in SLE is rare, and its main manifestations are short-term memory impairment, seizures, or psychological and behavioral abnormalities. Neurogenic pulmonary edema (NPE) is an acute pulmonary edema triggered by various central nervous system disorders, including spinal cord injury, subarachnoid hemorrhage, status epilepticus, and brain injury [2–4]. NPE is characterized by an acute fluid increase in the alveolar and interstitial pulmonary interstitium. Here, we report a case of NPSLE with NPE and AMPAR limbic encephalitis.

## Case presentation

**Fig. 1:**
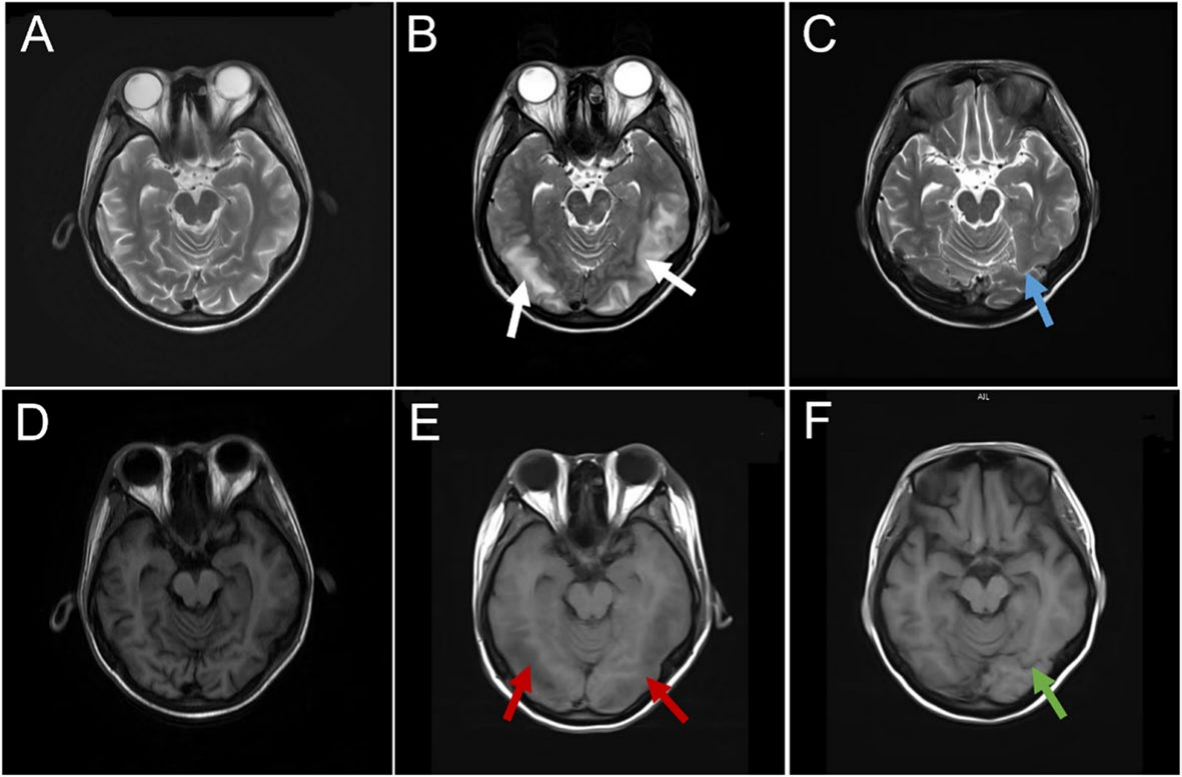
Magnetic resonance imaging brain findings. **A D** Before the onset of encephalopathy. **B E** Encephalopathy. MRI brain with T2 and T1 weighted images showed hyperintensity (white arrows) and hypointensity (red arrows) in bilateral temporoparietal subcortical areas, respectively. **C F** After treatment and reexamination. Hyperintensity (blue arrow) and hypointensity (green arrow) in temporoparietal subcortical areas disappeared

**Fig. 2:**
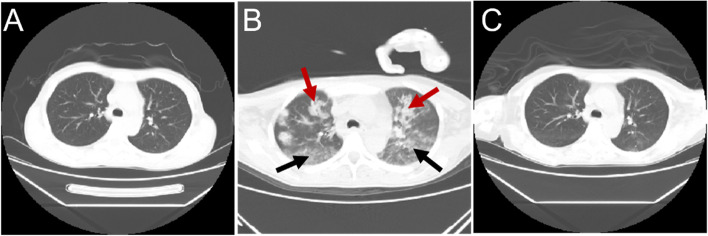
Chest computed tomography imaging findings. **A** Before the onset of encephalopathy. **B** Occurrence of neurogenic pulmonary edema. A CT axial scan of the chest showed multiple consolidations (red arrows) and ground-glass opacities (black arrows), mainly in the bilateral superior lobes. **C** After treatment and reexamination. HRCT showed obvious absorption of ground-glass opacities and consolidations

A 21-year-old woman with no relevant medical or family history of autoimmune disease was admitted to our hospital because of buccal rash, swelling and tenderness in the proximal interphalangeal joints of her hands. Other symptoms included morning stiffness, fatigue, dyspnea, numbness, hypoesthesia in the feet, dry mouth, and foamy urine. Laboratory examination showed that hemoglobin (107 g/L) and platelet (57 × 10^3^/µl) levels were decreased, while the erythrocyte sedimentation rate (59 mm/h) was accelerated. The titer of antinuclear antibody (ANA) was 1:1280. Anti-double-stranded DNA antibody, anti-Smith antibody, anti-cardiolipin antibody, anti-ribosomal P protein antibody, anti-nucleosome antibody, and anti-Sjögren’s syndrome A antibody were positive. Other antibodies were negative. She had hypocomplementemia (C3 (44.7 mg/dl) and C4 (5.48 mg/dl)), and total proteinuria at 24 h was 0.6 g. Her serum immunoglobulin G (IgG) level was 7870 mg/dL (normal range, 751 to 1560 mg/dL). Results of immunofixation electrophoresis of peripheral blood were negative. No abnormal signs were observed by magnetic resonance imaging (MRI) of the brain (Fig. 1A, D). Ultrasonic cardiogram (UCG) and chest high-resolution computed tomography (HRCT) (Fig. 2A) showed pericardial effusion. Based on the 2019 European League Against Rheumatism/American College of Rheumatology (EULAR/ACR) SLE classification criteria [5], 31 points were accumulated. After treatment with 80 mg/d methylprednisolone for 10 days and reduction to 40 mg/d for 5 days, 1.5 g/d mycophenolate mofetil and 0.4 g/d hydroxychloroquine for 10 days, the rash on both cheeks subsided; moreover, the patient’s other symptoms were relieved, and her condition gradually stabilized. On discharge, her IgG level had decreased to 2590 mg/dL.

Three days later, the patient had headache and dyspnea without fever or an epidemiological history of coronavirus disease-19. She suddenly experienced gnathospasmus, staring eyes, and five episodes of generalized tonic–clonic seizure. Therefore, she visited the emergency department. Physical examination showed grade II muscle strength of the limbs and decreased muscle tone, while no further abnormalities were found by other systematic neural examinations. MRI brain (Fig. 1B, E) revealed multiple T1 and T2 linear or patchy signal shadows in the bilateral subcortical area of the frontal, parietal, temporal, and occipital lobes; the white matter around the lateral ventricle; the basal ganglia thalamus area; the splenium of the corpus callosum; the brainstem; and the cerebellar hemisphere. Magnetic resonance angiography and venography of the head showed unremarkable findings. UCG showed a left ventricular ejection fraction of 57%, and pulmonary hypertension was present (45 mmHg). There was no previous underlying heart disease. Chest CT indicated multiple diffuse consolidations and ground-glass opacities in both lungs (Fig. 2B). Combined with her dyspnea, NPE was considered after inflammatory lesions and cardiogenic pulmonary edema were excluded. Lumbar puncture revealed elevated intracranial pressure (ICP) (260 mmH_2_O). There were increased protein levels (3.54 g/L) in cerebrospinal fluid (CSF) and cell counts, lactic acid, glucose, and chloride were all within normal limits. CSF cultures for bacteria and fungi were negative. The AMPAR2 antibody detected by cytometric bead array (CBA) was positive for both serum and CSF, whereas other autoimmune encephalitis-related antibodies were all negative in CSF or serum. Electrocardiography (EEG) revealed paroxysmal or sporadic medium amplitude theta activity and atypical sharp and slow waves. After treatment with methylprednisolone pulse therapy for three days (500 mg per day), the patient’s seizures stopped. However, she presented melena without nausea or vomiting several times. Abdominal CT showed a diverticulum in the horizontal part of the duodenum, peritoneal effusion, and pelvic effusion. The patient was treated with 40 mg/d Nexium, 0.6 mg/d octreotide, and 2 IU/d hemocoagulase for suspected gastrointestinal bleeding. Her stool returned to normal, and occult blood tests gradually became negative. No tumors were found by systematic examinations, including serum tumor marker analyses and chest and abdominal CT. The patient had epileptic seizures, and the results of CSF, MRI, and EEG analyses were abnormal. After ruling out infectious encephalitis, AMPAR encephalitis was diagnosed according to the diagnostic criteria of autoimmune encephalitis [6]. She was treated with 200 mg/d methylprednisolone, 500 ml/d glycerol fructose, 1.2 g/d sodium valproate, and 1.0 g/d levetiracetam. The dosage of methylprednisolone was decreased according to her clinical condition until it was maintained at 40 mg/d. No seizures occurred thereafter. MRI brain (Fig. 1C, F) and chest HRCT (Fig. 2C) showed obvious improvement after 5 days. One week later, repeated lumbar puncture revealed that the CSF protein levels (0.68 g/L) decreased and her ICP was normal. Reexamination of EEG showed a medium-amplitude alpha rhythm (9 Hz). On discharge, the patient’s IgG level had decreased to 1630 mg/dL. The patient has not yet been followed up.

## Discussion and conclusions

SLE is an autoimmune disease characterized by multiple organ damage predominantly manifesting in women of child-bearing age. The diagnosis of incipient SLE is mostly based on the 2019 EULAR/ACR classification criteria due to its higher sensitivity [7]. The serum ANA titer of our patient was 1:1280. She had thrombocytopenia (4 points), low C3 and C4 (4 points), proteinuria (4 points), and pericardial effusion (5 points). Anti-cardiolipin antibody (2 points) and anti-double-stranded DNA and anti-Smith antibodies (6 points) in serum were positive. Moreover, there was swelling, tenderness, and morning stiffness in the proximal interphalangeal joints of her hands (6 points). The patient’s score according to the 2019 EULAR/ACR classification criteria was 31 [5]. Based on her headache and epileptic seizures; positive anti-Smith, anti-cardiolipin, and anti-ribosomal P protein antibodies in serum; and signs revealed by brain MRI, NPSLE was diagnosed after ruling out other possible diseases.

Up to 50% of SLE patients develop respiratory system damage, such as pleurisy, interstitial lung disease, diffuse alveolar hemorrhage, pulmonary hypertension, and airway disease. However, NPE caused by lupus is rarely reported. There are no definitive diagnostic criteria for NPE, which is diagnosed only by excluding noncardiogenic pulmonary edema after a neurological injury. In our patient, there was a history of epileptic seizures and increased ICP before dyspnea onset. Chest CT showed diffuse multiple ground-glass density shadows in both lungs and decreased pericardial effusion. There was no thickening of the interlobular septum or bronchial vessels. UCG revealed a left ventricular ejection fraction of 57%, with little pericardial effusion. The ground-glass opacities in her lung improved significantly after 5 days of treatment. Moreover, her hemoglobin levels did not decrease over a short period. Therefore, NPE was considered; pulmonary hemorrhage and cardiogenic pulmonary edema were excluded from the diagnosis. Patients such as ours in the present case should be promptly treated with antiepileptic therapy, and hypoxemia and complications should be controlled.

Since the discovery of anti-N-methyl-D-aspartate receptor (NMDAR) encephalitis antibodies in 2007 [8], an increasing number of pathogenic autoimmune encephalitis antibodies, such as anti-gamma-aminobutyric acid receptor (GABAR) antibody, anti-leucine-rich, glioma-inactivated 1 (LGI1) antibody, anti-contactin-associated protein-like 2 receptor (CASPR2) antibody, AMPAR antibody, and immunoglobulin-like cell adhesion molecule 5 (IgLON5) antibody, have been recognized. In a study based on a population diagnosed with autoimmune encephalitis in Southwest China [9], 80.95% of patients were positive for antibodies against NMDAR, followed by anti-GABAR (7.41%), LGI1-related (4.76%), CASPR2-related (2.65%), NMDAR + GABAR (1.59%), LGI1 + CASPR2 (1.59%), NMDAR + CASPR2 (0.53%), and AMPAR2 + CASPR2 antibodies (0.53%). AMPAR antibody is a rare autoimmune encephalitis antibody in SLE. We searched PubMed and Embase for cases of AMPAR in SLE using the terms “systemic lupus erythematosus” and “AMPAR”. Only one reported case was found [10]. Both patients reported were female and experienced epileptic seizures before hospital admission. Their average age was 30. Bacterial and viral cultures of CSF were negative, and AMPAR antibody in CSF was positive in the two cases. Furthermore, no malignancy was found. Our patient attained remission by methylprednisolone pulse therapy and symptomatic treatment. The other patient attained remission after methylprednisolone pulse and plasma exchange therapy. Therefore, methylprednisolone pulse therapy or plasma exchange therapy alone or their combinations is recommended to relieve symptoms and improve the prognosis for SLE with AMPAR limbic encephalitis.

Although the pathogenesis of SLE-associated AMPAR limbic encephalitis is still unknown, AMPAR autoantibodies can be pathogenic. In humoral immunity, B cells are induced to differentiate into plasma cells via antigen stimulation and secrete large amounts of autoantibodies [11]. Specific antibodies for SLE include anti-double-stranded DNA and anti-Smith antibodies. We consider that activated B cells in SLE may also secrete autoimmune encephalitis antibodies, such as AMPAR, which can play a key role in the onset of limbic encephalitis. AMPAR-mediated excitatory postsynaptic currents may be reduced by increased internalization of AMPAR clusters and a decreased number of AMPAR antibodies on neuronal surfaces and synapses, resulting in signs and symptoms such as epileptic seizures and memory loss [12, 13].

To the best of our knowledge, autoimmune encephalitis may be a subclassification of NPSLE, but it has not yet been identified by the available literature. We consider that these clinical outcomes may be the collective result of the respective pathogenesis in our case. Further research is needed to explore the underlying mechanisms.

Therefore, when the polyclonal IgG titer becomes higher in patients with SLE, physicians need to maintain a high index of suspicion for the presence of new autoimmune antibodies. In particular, patients should be screened for autoimmune encephalitis antibodies when there is nervous system involvement. Although there is no clear guideline for the treatment of AMPAR encephalitis, methylprednisolone pulse therapy or plasma exchange therapy alone or their combinations is recommended to relieve symptoms and improve the prognosis for SLE with AMPAR limbic encephalitis.

In conclusion, NPE can present as a complication of NPSLE. And this report emphasizes that AMPAR2 antibodies may be produced in NPSLE patients, especially in those with high polyclonal IgG antibody titers. Early screening for encephalitis-related autoantibodies in NPSLE patients may improve prognosis by allowing timely appropriate treatment. More basic and clinical studies are required to confirm these observations and elucidate the pathogenicity of encephalitis-related autoantibodies in SLE patients.

## Data Availability

Not applicable.

## Abbreviations

SLE: Systemic lupus erythematosus
NPE: Neurogenic pulmonary edema
AMPAR: Anti-α-amino-3-hydroxy-5-methyl-4-isoxazolepropionic acid receptor
NPSLE: Neuropsychiatric lupus erythematosus
ANA: Antinuclear antibody
C3: Complement 3
C4: Complement 4
IgG: Immunoglobulin G
UCG: Ultrasonic cardiogram
MRI: Magnetic resonance imaging
CT: Computed tomography
EULAR/ACR: European League Against Rheumatism/American College of Rheumatology
HRCT: High-resolution computed tomography
ICP: Intracranial pressure
CSF: Cerebrospinal fluid
CBA: Cytometric bead array
EEG: Electrocardiography
NMDAR: N-methyl-D-aspartate receptor
GABAR: Gamma-aminobutyric acid receptor
LGI1: Leucine-rich glioma-inactivated 1
CASPR2: Contactin-associated protein-like 2
IgLON5: Immunoglobulin-like cell adhesion molecule 5
